# Special Issue “Mathematical Modeling of Viral Infections”

**DOI:** 10.3390/v10060303

**Published:** 2018-06-04

**Authors:** John M. Murray, Ruy M. Ribeiro

**Affiliations:** 1 School of Mathematics and Statistics, UNSW Australia, Sydney 2052, Australia; 2 Cancer Research Division, Cancer Council NSW, Woolloomooloo NSW 2011, Australia; 3 Theoretical Biology and Biophysics, Los Alamos National Laboratory, Los Alamos, NM 87545, USA; 4 Laboratorio de Biomatematica, Faculdade de Medicina da Universidade de Lisboa, 1649-028 Lisboa, Portugal

How an infection will progress in the body is dependent on myriad factors: the rate of spread of the agent, the immune response, what treatment may be applied, .... Clinically following the progression of the disease is limited to snapshots at discrete time points (longitudinally in an individual or in an in vitro/animal model if that is available, but most often from cross-sectional data over cohorts of individuals), and in many cases the data obtained are from a body compartment that is not the site of replication of the disease. We then infer disease progression by putting these discrete data together based on our biological knowledge of the systems involved. However, based on these snapshots, it is not straightforward to work out important disease properties, such as the rate of progression, pathogenicity, and treatment and immune response effects. Mathematical modelling provides a scientifically sound, reproducible method to describe the underlying dynamics that produce these data, as well as a means to investigate new scenarios, such as the effect of a new drug. Its impact has been demonstrated on a number of diseases, for example, contributing to a better understanding of the speed with which human immunodeficiency virus (HIV) replicates [1,2], the dynamics of different drug classes for HIV [3], the main mode of action of interferon in hepatitis C virus (HCV) [4], the effects of direct-acting antivirals in HCV [5], potential effects of the immune response [6], and many others [7,8,9,10,11].

This issue on mathematical modelling of viral infections covers a number of viruses: HCV [12,13,14,15], hepatitis B virus (HBV) [16,17], HIV [18], influenza [19], and even viruses that are used to combat cancer [20]. Moreover some of the modelling, although applied to specific diseases, has wider applications as they describe intracellular processes that are common to a number of infections [12,13,14]. Bingham et al. provide a description of the viral encapsidation process mediated by packaging signalling (PS) motifs, how this can result in a measure of viral fitness dependent on how well this packaging occurs, and then assess how targeting these conserved regions of the viral genome could produce HCV therapies that are less susceptible to viral escape than current direct acting agents [14]. The two works by Knodel and collaborators describe intracellular dynamics of HCV proteins and viral RNA over the endoplasmic reticulum (ER) and membranous webs within the cytoplasm [12,13], which will have wider application to other infections that use similar pathways for viral replication, assembly and export (potentially other *Flaviviridae* such as West Nile Virus, Dengue virus, and Zika virus).

The contribution of cellular proliferation and its subsequent role in elimination of HBV at acute infection is described by Goyal et al. [16]. Ganusov describes the escape rate of HIV against the CD8+ T cell response and how these estimates are affected by the time between viral sequence sampling [18]. Aston develops a new HCV model and considers its implications for treatment [15]. The impact of antiviral therapy was investigated by several groups. Rodriguez et al. determined the processes and state of chronic HCV infection that delineated the patterns of viral decay under therapy [17]. Cao and McCaw compare the two main types of influenza models and describe the best circumstances for each model’s use to predict the results of antiviral therapy [19].

Oncolytic viral therapy is a growing area of research. It uses viruses that preferentially replicate in cancer cells, either killing them as a direct result of viral replication, or through the induction of immune responses against the cancer. For those readers wanting an introduction to this important and growing field, the review by Santiago and collaborators provides an excellent coverage of the underlying biology of oncolytic viral therapy as well as the approaches to date in mathematical modelling in this area [20].

This issue also provides a good coverage of the different mathematical modelling approaches that can be used. Most of the models employed here are described by ordinary differential equations (ODEs). These have the advantage of a more straightforward implementation within numerical software, as well as requiring fewer parameters than agent-based models. For situations when both space and time elements are needed, such as in the work describing viral protein movement within HCV-infected hepatocytes [12,13], partial differential equations (PDEs) are the natural setting (to see how complex these dynamics can be and the geometry that can be incorporated into these models, the reader is directed to the movies in the Supplementary Material for these articles). Finally, where mutations must be tracked as is the case for the evolution of a viral quasi-species [14], a stochastic system of equations is necessary.

Finally, we note the interdisciplinary aspect of these works, with authorships covering hospitals, departments of medicine, biology, immunology, and oncology, high-performance computing groups, as well as mathematics departments. These are complex systems where the dynamics are not easy to understand and consequently require a mathematical description. But mathematics is only one aspect of successfully analysing the course of a disease. Any meaningful contribution requires an equal partnership with our biomedical collaborators. This Special Issue reflects this important partnership.
